# The past, present, and future of coral heat stress studies

**DOI:** 10.1002/ece3.5576

**Published:** 2019-08-22

**Authors:** Maha J. Cziesielski, Sebastian Schmidt‐Roach, Manuel Aranda

**Affiliations:** ^1^ Red Sea Research Center Division of Biological and Environmental Science and Engineering King Abdullah University of Science and Technology Thuwal Saudi Arabia

**Keywords:** acclimatization, adaptation, bleaching, climate change, corals, heat stress

## Abstract

The global loss and degradation of coral reefs, as a result of intensified frequency and severity of bleaching events, is a major concern. Evidence of heat stress affecting corals through loss of symbionts and consequent coral bleaching was first reported in the 1930s. However, it was not until the 1998 major global bleaching event that the urgency for heat stress studies became internationally recognized. Current efforts focus not only on examining the consequences of heat stress on corals but also on finding strategies to potentially improve thermal tolerance and aid coral reefs survival in future climate scenarios. Although initial studies were limited in comparison with modern technological tools, they provided the foundation for many of today's research methods and hypotheses. Technological advancements are providing new research prospects at a rapid pace. Understanding how coral heat stress studies have evolved is important for the critical assessment of their progress. This review summarizes the development of the field to date and assesses avenues for future research.

## 1 INTRODUCTION

In 2016, the longest El Niño event recorded to date resulted in mass bleaching events of coral reefs worldwide (Claar, Szostek, McDevitt‐Irwin, Schanze, & Baum, 2018). Increases in sea surface temperatures caused tropical corals to experience thermal stress beyond their tolerance (Lough, Anderson, & Hughes, 2018). When corals experience extreme and prolonged heat stress, their symbiotic relationship with the algae of the family Symbiodiniaceae (previously classified as the genus Symbiodinium (LaJeunesse et al., 2018)) is affected and can ultimately break down, a process widely known as coral bleaching (Hughes et al., 2003). The algae provide most of the energetic requirements of the coral host (Muscatine, Falkowski, Porter, & Dubinsky, 1984), enabling them to effectively calcify and become the foundation of modern reefs. Symbiodiniaceae loss within the host results in energetic deficit that can eventually lead to the coral's death. In the last two decades, the observed loss of symbionts and bleaching response to heat stress has been extensively studied. Yet, the underlying molecular mechanisms of host and symbiont heat stress response are still not fully understood (Blackstone & Golladay, 2018). The following review discusses the development of this field, from initial observations in the early 1900s to today's modern uses of ‐omics tools.

A century ago, the importance of symbionts to corals, and hence the biological significance of bleaching, was vastly underappreciated. Early hypotheses on the nutritional relationship between corals and algae varied (Boschma, 1925; Mayer, 1915), but the concept of photosynthetic product exchange from symbiont to host was quickly acknowledged (Gardiner, 1931). The complexity of this relationship was recognized by perceiving the coral as an ecological unit—a holobiont (Knowlton & Rohwer, 2003; Odum & Odum, 1955). Toward the turn of the 20th century, a clearer understanding of coral stress responses was developed. Early research showed declines in symbiont density coincided with the loss of chlorophyll pigments, decreased photosynthesis rate, and increased respiration in the host. Additionally, coral protein, lipid, carbohydrates, and calcification rate were also reduced as a direct consequence of symbiosis breakdown (Goreau, 1959; Muscatine & Lenhoff, 1965; Muscatine, 1967; Coles, Jokiel, & Lewis, 1976; GGlynn, 1985, Glynn, 1993; Hoegh‐Guldberg & Smith, 1989; Porter, Friedland, Wojnarowska, & Ledingham, 1989; Glynn & D'Croz, 1990; Jokiel & Coles, 1990). Interest in coral bleaching and temperature stress ignited in the late 1990s when the first evidence of mass bleaching was recorded after a series of El Niño events.

The El Niño Southern Oscillation (ENSO) incident of 1998 was the first mass bleaching event recorded by the Hotspot program of the US National Oceanic and Atmospheric Administration (Liu et al., 2014; Strong, Barrientos, & Duda, 1996). The program predicted, weeks in advance, which geographical regions would experience bleaching due to increased sea surface temperatures. These findings supported the growing evidence that climate change was having severe impacts on marine ecosystems, fueling the need for a better understanding of coral symbiotic relationships. The cumulative observations of the ENSO events in the 1980s and 1990s revealed that the consequences of temperature stress and coral bleaching were much greater than imagined. These consequences include increased mortality, decreased reproduction, reduced reef productivity, and changes in community structure (Hoegh‐Guldberg, 1999). Nonetheless, growing reef monitoring efforts have revealed potential adaptation and acclimatization strategies of corals and their symbionts, providing hope for their survival under a changing climate. This has moved scientists into a new avenue of research and provided a novel outlook on coral conservation.

The growing recognition of coral reefs' high socioeconomic importance (Black & Bloom, 1984; Carte, 1996; Hoegh‐Guldberg, 1999; Jameson, McManus, & Spalding, 1995) has propagated interest in understanding reef systems, their functions, and how to aid in their survival. Our understanding of coral–algal symbiosis and environmental stress responses has progressed significantly, with rapid technological advancement enabling further insight into the complex dynamics of this relationship. While physiological measurements were, and are, fundamental in understanding coral's responses to stressors, understanding underlying molecular mechanisms of coral symbiosis, acclimatization, and adaptation is critical if we want to aid coral reef survival. This review assesses coral heat stress studies and their development over time. Based on the past and present progress, we provide suggestions on the future directions of this field.

## 2 WHERE ARE WE NOW?

The studies of the early 1990s revealed the importance of Symbiodiniaceae to the overall thermotolerance of the holobiont. Symbiodiniaceae associations assist the holobiont through the following: plasticity in response to temperature and irradiance (Lesser, 1997; Lesser, Stochaj, Tapley, & Shick, 1990), downregulation of photosynthesis (Brown, Ambarsari, et al., 1999), shuffling of symbiont clades in the host to those better adapted to the stressors (Buddemeier & Fautin, 1993; Hoegh‐Guldberg, Jones, Ward, & Loh, 2002; Rowan, Knowlton, Baker, & Jara, 1997), and xanthophyll cycling (Brown, Dunne, Ambarsari, Le Tissier, & Satapoomin, 1999). Studies on both partners have increasingly shown the inherent complexity of the holobiont stress response; coral and algae determine holobiont tolerance (R. O. B. Baker, Starger, McClanahan, & Glynn, 2004; Iglesias‐Prieto & Trench, 1994; Rowan et al., 1997; Rowan & Powers, 1991). However, thermotolerance is variable between and within coral species. Understanding mechanisms and indicators of thermal susceptibility has thus been a central focus of coral heat stress studies. The early development of stress response biomarkers enabled a monitoring system and provided a basis for comparison.

### 2.1 Proteins: the first molecular insights

The first biomarkers to be confidently established in coral heat stress studies were heat‐shock proteins (HSPs). Prior heat stress studies in other cnidarians, such as *Hydra* (Bosch, Krylow, Bode, & Steele, 1988), *Anemonia viridis* (Miller, Brown, Sharp, & Nganro, 1992), and the jellyfish *Aurelia aurita* (Black & Bloom, 1984), showed the presence and the increased abundance of HSPs in thermal stress response. The first 70‐kDa HSP homologue in corals was characterized in *Goniopora dijboutiensis* (Sharp, Miller, Bythell, & Brown, 1994). Additional studies on other coral and anemone species extended the repertoire of HSP homologues, establishing them as viable biomarker candidates (Black, Voellmy, & Szmant, 1995; Branton, MacRae, Lipschultz, & Wells, 1999; Wiens et al., 2000).

Thermal stress has also been shown to increase reactive oxygen species (ROS) in corals and algal symbionts, consequently triggering antioxidant mechanisms. Antioxidant pathways control cell‐level toxicity and, thus, cellular stress and damage during stress events. Antioxidant‐related protein's ability to maintain ROS levels at nontoxic levels made them interesting targets for biomarker development. Although proteins such as superoxide dismutase (SOD), catalase (CAT), and ascorbate peroxidase (ASPX) had been shown to play critical roles in coral's antioxidative stress response (e.g., Dykens, Shick, Benoit, Buettner, & Winston, 1992; Lesser & Garcia, 1997; Lesser, 1996; Lesser et al., 1990), their use as biomarkers only developed in the early 2000s.

Proteins known to be important for antioxidant mechanisms such as B‐crystallin, copper/zinc SOD (Cu/ZnSOD), manganese SOD (MnSOD), ubiquitin, lipid peroxide (LPO), and total glutathione (GSH) were detected and measured in *O. faveolata* (Downs, Mueller, Phillips, Fauth, & Woodley, 2000). Proteins showed a higher abundance in heat‐ and light‐stressed corals under laboratory conditions as well as during natural bleaching events (Downs et al., 2000; Lesser, 1996). These findings confirmed the coral host was experiencing oxidative stress, as a consequence of the symbionts' damaged photosystem II (PSII). Additionally, ROS was shown to compromise host cell integrity and consequently induce bleaching (Hoegh‐Guldberg, 1999). Follow‐up studies showed that oxidative stress caused by symbionts also triggered host nitric oxide production through the activation of NF‐kB, leading to cell death and bleaching (Perez & Weis, 2006).

An increased repertoire and understanding of these biomarkers enabled the comparison of stress responses between coral species. Interspecies comparisons revealed ambient HSP70 levels differed between two species and multi‐HSP expression was an indicator of improved thermal response (Robbart, Peckol, Scordilis, Curran, & Brown‐Saracino, 2004). However, variations in thermal tolerance were observed not only between species but also within species and even within single colonies (Brown, Downs, Dunne, & Gibb, 2002; Brown, Dunne, Goodson, & Douglas, 2000; Cook, Logan, Ward, Lcukhurst, & Berg, 1990; Goreau & Macfarlane, 1990; Jokiel & Coles, 1990). A single coral colony can grow into a large structure where parts of the colony may experience differences in competition, light regime, and temperature. Thus, single large corals can often experience a variety of conditions. Use of both HSP and oxidative stress‐related biomarkers showed that large coral colonies had significant differences in thermal stress experience across sections (Brown et al., 2002). Further studies focused on understanding tissue‐specific expression of biomarkers (Richier et al., 2003), their general characterization (Plantivaux et al., 2004), and potential role in symbiosis (Richier, 2005). These studies contributed to the general understanding of selected biomarkers' functions and their variabilities in corals.

Protein biomarkers have facilitated a deeper understanding of the underlying mechanisms driving tolerance variations observed within and between species. Comparing response patterns and establishing viable biomarkers based on protein abundances became a well‐accepted method in coral heat stress studies. However, the use of protein‐based analyses changed when new technological advancement enabled expression analysis of various mRNA transcripts, simultaneously.

### 2.2 The rise of transcriptomics

Understanding of the coral heat stress response experienced a significant leap forward in 2005, when the first coral cDNA microarray was published (Edge, Morgan, Gleason, & Snell, 2005), pioneering transcriptomics in coral research. Microarrays were the first form of transcriptome expression studies in which reverse‐transcribed and fluorescently labeled mRNAs (cDNA) were hybridized to known complementary DNA targets and quantified based on their fluorescent intensity. The cDNA fragments, or expressed sequence tags (EST), on the first coral microarray represented 32 different cDNAs from *Acropora cervicornis* and *Orbicella faveolata,* which showed responses to different environmental stressors in prior studies. For the first time, pathways related to ribosomal RNA (protein biosynthesis), ferritin, thioredoxin (oxidative stress), and carbonic anhydrase (skeletal growth) were shown to have roles in the coral heat stress response. This basic array was quickly superseded by a more extensive version, with 10,368 features from *Anthopleura elegantissima* (Rodriguez‐Lanetty, Phillips, & Weis, 2006). This new array was used to examine transcriptome‐wide responses to temperature and UV stress (Richier, Rodriguez‐Lanetty, Schnitzler, & Weis, 2008). The study confirmed previous observations by Edge et al. (2005) and provided insight into new pathways involved in heat stress response and stress mitigation, such as actin (cytoskeleton structure), ferritin (oxidative stress), ribosomal proteins (protein biosynthesis), and Rab7 (membrane trafficking). With the combined efforts of these initial microarray studies, genes were identified from a variety of cellular pathways, providing evidence that the breakdown of symbiosis was a result of multiple interactions (Dunn, Schnitzler, & Weis, 2007). Nonetheless, microarrays had their limitations in that only a number of known sequences could be studied. The development of next‐generation sequencing (NGS), which allowed the total mRNA content of an organism (the transcriptome) to be sequenced, overcame these shortcomings and provided new insight into the molecular layers of organisms.

**Figure 1:**
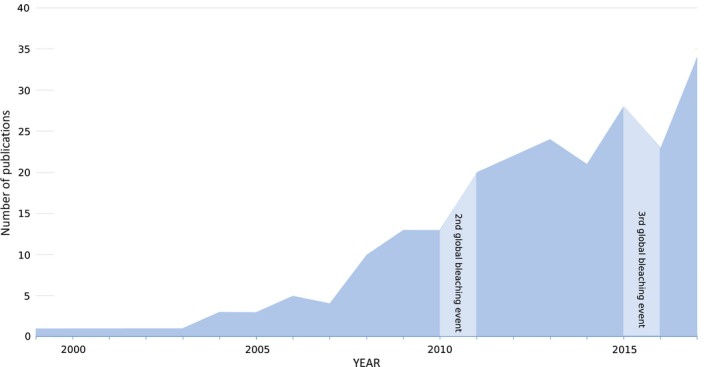
Number of publications on cnidarian heat stress response using ‐omics tools. Publication records with keywords were recorded from Web of Knowledge and plotted according to year of publication (https://apps.webofknowledge.com/). Since the development of the first cnidarian microarray in 2005 and technological advancement of various molecular sequencing platforms, the application of ‐omics tools has increased steadily. Keywords used to determine studies were separated into independent variables (or) within three categories donated by (and). The keywords used were as follows: “heat stress or temperature stress or thermal stress” and “coral or anemone or anthozoan” and “gene expression or transcriptome or transcriptomics or proteomics or genomics or genome”

Whole‐genome, transcriptome, and proteome sequencing, collectively known as ‐omics tools, have opened the field to new possibilities, hypotheses, and information regarding heat stress resilience of coral holobionts. The possibility of whole‐mRNA sequencing propelled transcriptome studies in a variety of corals (Meyer et al., 2009; Schwarz et al., 2008; Traylor‐Knowles et al., 2011) as well as in Symbiodiniaceae species (Barshis, Ladner, Oliver, & Palumbi, 2014; Bayer et al., 2012; Rosic et al., 2015). Though not comprehensively discussed in this review, a growing number of studies are applying proteomics in cnidarians (Barneah, Benayahu, & Weis, 2006; Cziesielski et al., 2018; Drake et al., 2013; Oakley et al., 2016; Ramos‐Silva et al., 2013). Omics methods were previously only utilized in a handful of studies, but are currently one of the most common tools applied in the field (Figure 1) and thus the primary focus of the following sections.

### 2.3 Transcriptomics and coral's molecular stress response mechanisms

**Figure 2:**
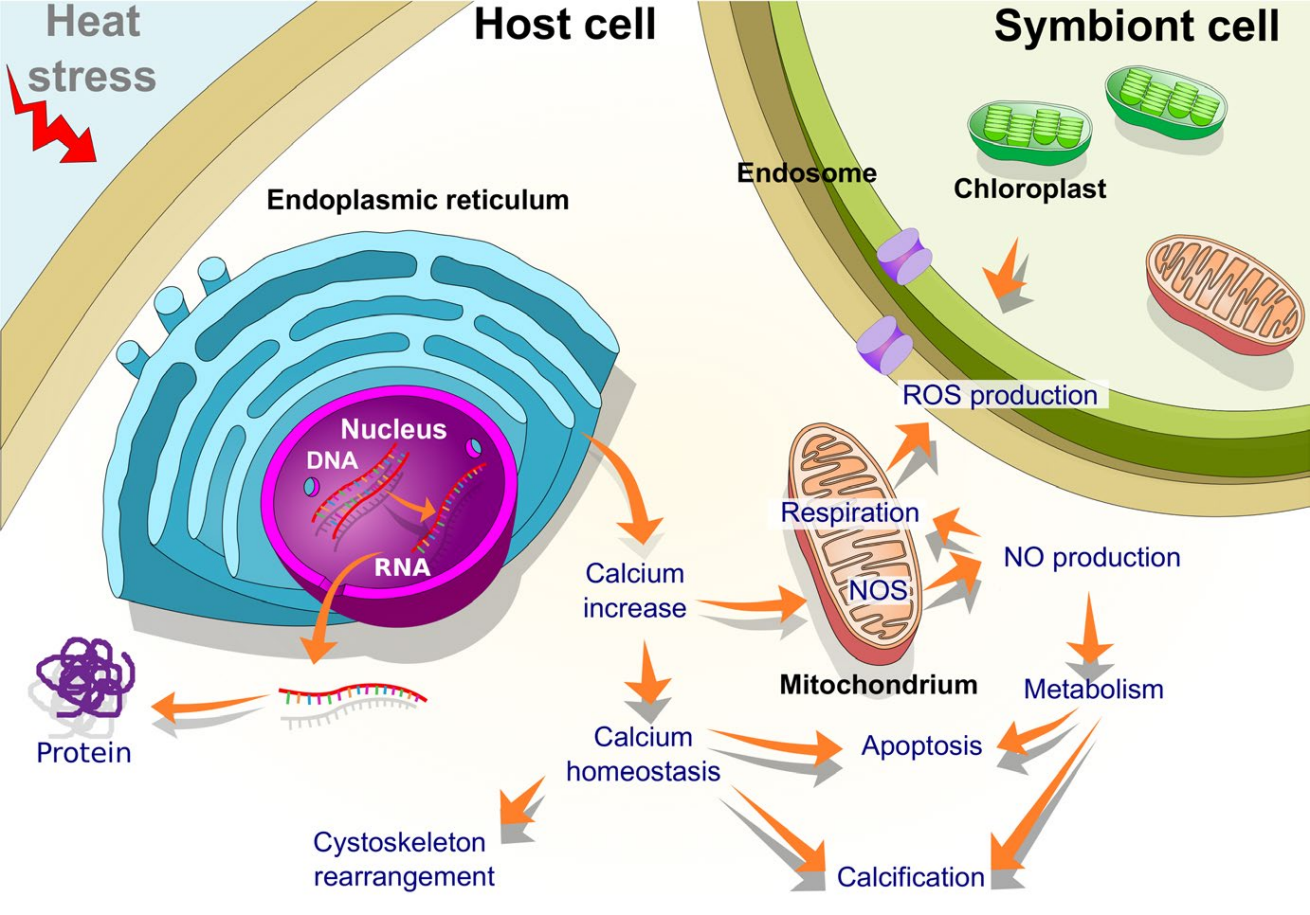
Summary of coral heat stress responses. Increasing temperatures trigger calcium release from the endoplasmic reticulum, which leads to various changes in cell function (e.g., cytoskeleton rearrangement, cell adhesion disruption) through disruption of calcium homeostasis. Meanwhile, the metabolic rate is also increased, causing an increase not only in reactive oxygen species (ROS) but also in nitric oxide (NO). Consequently, oxidative stress from ROS and NO is experienced by the coral, which can ultimately lead to apoptosis or necrosis. Symbiodiniaceae have their own temperature tolerance and responses but also produce ROS under stress, which can leak into the host and exacerbate oxidative stress

Applications of transcriptomics have rapidly expanded, and with it our understanding of coral molecular stress responses. Studies on important reef‐building species, such as *O. faveolata* (DeSalvo et al., 2008) and *Acropora palmata* (DeSalvo, Sunagawa, Voolstra, & Medina, 2010), have revealed an assortment of crucial heat stress genes in cnidarians, such as peroxidasin, C/EBP, EF‐hand, and calmodulin. The use of microarrays to compare heat stress responses provided the first validations of the accuracy at which biomarkers could be confidently used across species. Biomarkers such as NF‐kB, caspase‐3, TNF receptor‐associated factor 3 (TRAF3), and Cu/ZnSOD are commonly increased during heat stress across species (DeSalvo, Sunagawa, Fisher, et al., 2010). Extensive research over the years and the standardization of transcriptomics not only lead to a better understanding of thermotolerance mechanisms but also revealed common patterns. Temperature‐stressed cnidarians experienced (a) increased HSP expression, (b) increased antioxidant expression, (c) decreased Ca^2+^ homeostasis, (d) restructured ECM, (e) rearrangement of actin cytoskeleton, (f) decreased ribosomal protein expression, and (g) pro‐apoptotic responses (Abrego, Ulstrup, Willis, Van, & Oppen, 2008; Barshis et al., 2013; DeSalvo, Sunagawa, Fisher, et al., 2010; Fitt et al., 2009; Kenkel, Meyer, & Matz, 2013; Maor‐Landaw & Levy, 2016) (Figure 2). These response patterns are not only limited to adult colonies, but heat stress studies on larvae and larval development have also shown similar cellular responses (Negri, Marshall, & Heyward, 2007; Polato et al., 2010; Portune, Voolstra, Medina, & Szmant, 2010; Rodriguez‐Lanetty, Harii, & Hoegh‐Guldberg, 2009; Voolstra et al., 2009). The consistency in the expression of critical heat stress pathways showed the existence of common response mechanisms across cnidarians. The shared responses, and selected associated genes, can be considered the core cnidarian heat stress response (Cziesielski et al., 2018).

### 2.4 More than just a coral: understanding the holobiont's thermotolerance

The expansion of ‐omics tools has allowed functional insight into thermotolerance and the proposed role of the symbionts in coral bleaching resilience, as Symbiodiniaceae strain‐specific thermal resistance has repeatedly indicated improved host stress response (Berkelmans & van Oppen, 2006; Howells, Abrego, Meyer, Kirk, & Burt, 2016; Oliver & Palumbi, 2011; Palumbi, Barshis, Traylor‐Knowles, & Bay, 2014; Pinzón et al., 2015; Polato et al., 2010; Silverstein, Cunning, & Baker, 2015). Symbiodiniaceae research continues to uncover complex interactions and response mechanisms, often relating to oxidative stress (ROS and NO stress (Abrego et al., 2008; Bouchard & Yamasaki, 2008; DeSalvo, Sunagawa, Fisher, et al., 2010; Hume et al., 2015; Hume et al., 2016; Levin et al., 2017; Littman, Bourne, & Willis, 2010; Middlebrook, Hoegh‐Guldberg, & Leggat, 2008)), nutrient exchange, and metabolic compatibility (Davy, Allemand, & Weis, 2012; Rädecker et al., 2018; Suggett, Warner, & Leggat, 2017; Wiedenmann et al., 2012).

Although research endeavors have mainly focused on the cnidarian host and algal symbiont, there has also been growing recognition of another important holobiont component: the microbiome. Prior research on coral microbiomes were primarily associated with disease response and immunity (Bourne & Munn, 2005; Cooney et al., 2002; Pantos et al., 2003; Rosenberg, Koren, Reshef, Efrony, & Zilber‐Rosenberg, 2007). Recent studies have provided sufficient evidence that microbiomes could contribute to the holobiont's temperature tolerance and potentially provide resilience (Diaz et al., 2016; Glasl, Herndl, & Frade, 2016; Littman, Willis, & Bourne, 2011; Reshef, Koren, Loya, Zilber‐Rosenberg, & Rosenberg, 2006; Thurber et al., 2009; Ziegler, Seneca, Yum, Palumbi, & Voolstra, 2017). Today, the probiotic theory of corals (Reshef et al., 2006), describing the dynamic relationship between symbiotic microorganisms and environmental conditions to create the most advantageous holobiont, is an expanding focus of coral heat stress studies.

Evidently, thermal resilience cannot solely be attributed to only one of the members of the holobiont. However, since this review focuses on the cnidarian host, we have only briefly touched on the other members. Growing evidence suggests that the host genotype is capable of local adaptation and acclimation (Bellantuono, Hoegh‐Guldberg, & Rodriguez‐Lanetty, 2012; Hawkins, Krueger, Wilkinson, Fisher, & Davy, 2015). Host genotype response variability is particularly important, as studies have shown improved tolerance in corals with previous exposure, indicating that resilience may be heritable (Dixon et al., 2015; Howells et al., 2016). The concept of pre‐exposure has gained increasing attention in recent years, as it may provide a crucial platform for coral survival in light of global change.

### 2.5 Learning from experience: Life history and pre‐exposure to stress provide platforms for coral resilience

Environmental history can significantly impact coral's response to elevated temperatures and their overall tolerance to extreme events (Hawkins & Warner, 2017; Krueger et al., 2017; Rivest, Kelly, DeBiasse, & Hofmann, 2018). The hypothesis that prior heat exposure could improve a coral's response to follow‐up stress events was proposed early on (Coles & Jokiel, 1978; Jokiel & Coles, 1977; Middlebrook, Anthony, Hoegh‐Guldberg, & Dove, 2010; Middlebrook et al., 2008). Experiencing sublethal doses of thermal stress can provide a new acclimated baseline for subsequent stress events, by setting in place physiological and molecular mechanisms crucial in heat stress response (Ainsworth et al., 2016; Berry & Gasch, 2008). These observations indicate corals' potential to acclimatize to new environmental conditions. A recent large‐scale observational study, based on a model of the Great Barrier Reef's sea surface temperatures (SST), showed that prestress events occurred prior to the main stress, serving as a physiological preparation (Ainsworth et al., 2016). This study further validated its observations with in situ heat stress studies on *Acropora aspera,* reporting significant differences in gene expression profiles between pre‐exposure and control conditions. Not only can preconditioned corals show transcriptional differences (Barshis et al., 2013; Bellantuono, Granados‐Cifuentes, Miller, Hoegh‐Guldberg, & Rodriguez‐Lanetty, 2012; Bellantuono, Hoegh‐Guldberg, et al., 2012), but they also have the capacity to maintain higher Symbiodiniaceae densities under stress (Bay & Palumbi, 2015; Palumbi et al., 2014). Additionally, some studies suggest that preconditioned corals could potentially utilize the same genes but achieve larger magnitude in gene expression change (Bay & Palumbi, 2015; Kenkel & Matz, 2016).

A growing number of studies suggest that epigenetic mechanisms may play critical roles in the acclimatization process of corals. Epigenetic modifications such as DNA methylation, the addition of methyl groups to specific sites on a genome, and histone modifications, packaging proteins that bind DNA to condense it into chromosomes, are currently severely understudied in corals (Eirin‐Lopez & Putnam, 2019). Differential expression of transcripts can be a consequence of changes in DNA methylation distribution in response to stressors (Dixon et al., 2015). Hence, changes in DNA methylation sites have been linked to transcriptional plasticity, which may facilitate response mechanisms to a previously encountered stressor (Dimond & Roberts, 2016; Liew et al., 2018; Putnam, Davidson, & Gates, 2016). Although complex gene regulation through histone modifications is conserved in cnidarians (Schwaiger et al., 2014), knowledge regarding the role of histones in coral acclimatization and adaptation is lacking. Epigenetics and preconditioning appear to be promising mechanisms for coral adaptation and survival. However, it requires a significantly greater understanding before these mechanisms can be successfully utilized to their full potential.

## 3 WHERE SHOULD WE GO?

Potential directions for future work are plentiful. Living in the ‐omics age also means continuous possibilities to venture into new research avenues. Nonetheless, considering the past and present progress of coral heat stress studies, certain subjects stand out, which will require significant attention if we hope to increase our understanding of coral thermotolerance, and aid in their survival.

### 3.1 Reference genomes and model organisms

High‐quality reference genome assemblies are the key to informative molecular genetic studies. In general, the availability of reference genomes will also promote venturing into new fields of interest such as comparative genotyping and epigenetics. *Hydra magnipapillata* (Chapman et al., 2010) and *Nematostella vectensis* (Sullivan et al., 2006) genomes have been stable reference points, providing many evolutionarily conserved cnidarian genes that could be utilized in transcriptomic studies. However, unlike corals, neither of these two cnidarians associate with endosymbionts of the family Symbiodiniaceae. Thus, it was necessary to develop high‐quality genomes for corals. The first coral genome of *Acropora digitifera* (Shinzato et al., 2011) initiated studies on conserved mechanisms and an estimation of the depth of divergence between corals and other cnidarians. However, *A. digitifera* lies in the complex clade of the scleractinians, thus only representing a portion of corals. Phylogenetic analyses of robust and complex corals indicated that these clades separated at least 245 mya (Simpson, Kiessling, Mewis, Baron‐Szabo, & Müller, 2011), leaving enough time for divergence and the evolution of clade‐specific traits and adaptations. For some time, there was a severe lack in robust clade coral genomes that was only recently remedied. The *Stylophora pistillata* genome provided the first genomic resource for the robust clade (Voolstra et al., 2017). Reference genomes of a diverse range of corals will provide further insight into their biology and enable the development of new molecular tools. Yet, only four fully sequenced genomes are currently available (*Acropora digitifera* (Shinzato et al., 2011), *Acropora millepora* (Ying et al., 2019), *Pocillopora damicornis* (Cunning, Bay, Gillette, Baker, & Traylor‐Knowles, 2018), and *Stylophora pistillata* (Voolstra et al., 2017)). The Reef Future Genomics Consortium (Voolstra et al., 2015) recognized the urgency of this problem. They defined a set of 10 coral species for which to investigate physiological differences and identified a framework of molecular datasets that are anticipated to provide new insight into coral's adaptive capabilities. Although development of new reference genomes is required, the progress of these needs to occur simultaneously with the optimization and development of genetic tools for existing sequenced genomes.

While there is a strong interest in making more coral genomes available (Liew, Aranda, & Voolstra, 2016), there is also the proposition of a coral model organism (Baumgarten et al., 2015), such as the small sea anemone *Aiptasia pallida (*sensu* Exaiptasia pallida)*. Having a model organism allows stronger international efforts to gain an integrative understanding of cnidarian biology by allowing studies to be combined and directly comparable. Additionally, working on the same established model organism could speed up the development of molecular tools.

### 3.2 Integrative analysis and secondary validation

There is no doubt that transcriptomics has provided invaluable insight into stress response in corals. However, the main limitation of transcriptomics is that it does not necessarily reflect the physiological response. Hence, coral heat stress research requires molecular and physiological measurements to be incorporated together to fully understand thermotolerance.

Formation of mRNA is only the first step in a long chain of regulatory mechanisms leading to the final protein (Baumgarten et al., 2018). Through these, a single mRNA can potentially translate into thousands of proteins and be controlled by a number of regulatory mechanisms at posttranscriptional and posttranslational levels. The analysis of mRNA is seldom a representation of the protein content in the organism, which is frequently reflected in the poor correlation reported between mRNA and protein expression (Cziesielski et al., 2018; Griffin et al., 2002; Lee et al., 2003).

Discrepancies between the transcriptome and proteome cause concern not only for interpretation of data but also for the development of new biomarkers. Whereas previous biomarkers were established based on protein extraction and identification, current markers are suggested predominantly on the presence of mRNA. In particular, combined transcriptome–proteome approaches have the capacity for complementing one another (Seliger et al., 2009), allowing for data integration to provide a better understanding of a system or its current situation (Gomez‐Cabrero et al., 2014). A narrow assortment of papers utilizes proteomic analysis to elaborate on fundamental coral biology such as symbiosis, larval development, and calcification (Barneah et al., 2006; Drake et al., 2013; Oakley et al., 2016; Ramos‐Silva et al., 2013), but work related to proteomic stress response in corals is sparse (Cziesielski et al., 2018; Matthews et al., 2017; Weston et al., 2015). While technology and analytical tools are quickly progressing, large‐scale studies on proteins are not as feasible as for nucleic acids (Graves & Haystead, 2002). Secondly, the application of proteomics is not as standardized as that for mRNA‐seq. Methods are being developed for integrative analysis of multi‐omics data to illustrate more comprehensive pictures of the molecular systems (Bersanelli et al., 2016). Achieving data integration is a difficult challenge that has not been simplified by the rapidly increasing amount of data.

With the growing use of ‐omics tools, it is important that the targeted biological question should drive the use of these tools instead of embarking on a frenzy of large‐scale sequencing. Those that choose to focus their work on ‐omics should consider physiologically validating their observations. One omic layer might not represent the other, but if the phenotype does not support molecular findings, a reassessment of conclusions drawn may be advisable. Additionally, ‐omics users should consider creating clear hypotheses that can be incorporated and tested by physiologists or others. The important lesson learned from such tools is that identifying long lists of genes and proteins often generates more questions, which, when fully utilized, can lead to new research and progression in the field.

### 3.3 The holistic holobiont

The term coral holobiont comprises the totality of the coral symbiotic relations including, but not limited to, endosymbiotic zooxanthellae, bacteria, archaea, viruses, and fungi. All are part of what collectively is termed the microbiome, and each plays a role in the response of the holobiont. Research can often be targeted to a specific symbiont of interest. As much as we need to simplify the system into individual parts in order to confidently discern the role of each player, however, we must also remember that it is intricately connected. Ultimately, the goal is to understand system requirements and describe the relationship between each component to unveil the mechanisms of thermal tolerance.

Cnidarian host and Symbiodiniaceae temperature response are often pursued as separate fields. Recent studies have increasingly been combining coral and Symbiodiniaceae responses in their hypothesis testing. A growing understanding of the metabolic host–symbiont relationship has encouraged the use of methods that allow the measuring of these dynamics, such as metabolomics (Cui et al., 2019; Matthews et al., 2017) or NanoSIMS (Krueger et al., 2018). These have shown that the metabolic balance between the two partners is not only sensitive to environmental stressors but specifically fine‐tuned (Cui et al., 2019; Li et al., 2018; Matthews et al., 2017; Nielsen, Petrou, & Gates, 2018). The coral microbiome has also been shown to significantly impact the thermal stress response mechanisms of the coral as well as on their symbionts (Littman et al., 2010; Pogoreutz et al., 2018; Ziegler et al., 2017). Evidently, interactions between the different partners of the holobiont are extremely important to consider when attempting to understand the overall response. Our understanding of coral temperature and bleaching tolerance hypotheses increasingly acknowledges the difficulty of discerning the role of one partner from the other.

Targeting the complexity of the individual components of the holobiont was recognized by the ReFuGe Consortium (Voolstra et al., 2015), and explores the sequencing of the various components of the coral holobiont. Integrative approaches are required to efficiently compare and contrast not only different molecular layers, but also the response and interaction of different members of the system. This interplay is particularly important in light of current aims at aiding corals in surviving rapid climate changes.

## 4 CONCLUSION

Realization of the economic benefit of reef systems coupled with undeniable evidence of climate change impacts has fueled the field of coral heat stress studies. The emergence of new research methods such as transcriptomics has led to a continuous expansion of knowledge in the field. However, rapid advancements in technology perpetuate the increase in data generated, which may distract from developing mechanistic understanding. In these times, it is important to reflect upon the path that research endeavors have taken, build upon these, and expand in directions suitable for the long‐term goal of understanding how we may possibly ensure a future for coral reefs.

Gathering knowledge on the intricate system of the coral holobiont and combining the different parts to allow deeper insight into the overall response mechanisms will require a collaborative effort. Only then can we hope to find successful ways to aid corals in acclimatizing and adapting to the rapidly altering environment.

## CONFLICT OF INTEREST

None declared.

## AUTHOR CONTRIBUTIONS

MJC conceived the idea and wrote the first draft of the manuscript. SSR designed the figures. MA provided guidance and structure. All authors read and approved the manuscript.

## Data Availability

There are no data to be accessed or deposited.
